# Open monitoring meditation reduces the involvement of brain regions related to memory function

**DOI:** 10.1038/s41598-018-28274-4

**Published:** 2018-07-02

**Authors:** Masahiro Fujino, Yoshiyuki Ueda, Hiroaki Mizuhara, Jun Saiki, Michio Nomura

**Affiliations:** 1 Graduate School of Education, Kyoto University, Yoshida-Honmachi, Sakyo-ku, Kyoto-shi, Kyoto, 606-8501 Japan; 2 Kokoro Research Center, Kyoto University, 46 Shimoadachi-cho, Yoshida, Sakyo-ku, Kyoto-shi, Kyoto, 606-8501 Japan; 3 Graduate School of Informatics, Kyoto University, Yoshida-Honmachi, Sakyo-ku, Kyoto-shi, Kyoto, 606-8501 Japan; 4 Graduate School of Human and Environmental Studies, Kyoto University, Yoshida-Honmachi, Sakyo-ku, Kyoto-shi, Kyoto, 606-8501 Japan; 5 Japan Society for the Promotion of Science, 5-3-1, Kojimachi, Chiyoda-ku, Tokyo, 102-0083 Japan

## Abstract

Mindfulness meditation consists of focused attention meditation (FAM) and open monitoring meditation (OMM), both of which reduce activation of the default mode network (DMN) and mind-wandering. Although it is known that FAM requires intentional focused attention, the mechanisms of OMM remain largely unknown. To investigate this, we examined striatal functional connectivity in 17 experienced meditators (mean total practice hours = 920.6) during pre-resting, meditation, and post-resting states comparing OMM with FAM, using functional magnetic resonance imaging. Both FAM and OMM reduced functional connectivity between the striatum and posterior cingulate cortex, which is a core hub region of the DMN. Furthermore, OMM reduced functional connectivity of the ventral striatum with both the visual cortex related to intentional focused attention in the attentional network and retrosplenial cortex related to memory function in the DMN. In contrast, FAM increased functional connectivity in these regions. Our findings suggest that OMM reduces intentional focused attention and increases detachment from autobiographical memory. This detachment may play an important role in non-judgmental and non-reactive attitude during OMM. These findings provide new insights into the mechanisms underlying the contribution of OMM to well-being and happiness.

## Introduction

Mindfulness meditation is a form of attentional control training by which individuals develop the ability to direct and maintain attention towards the present moment^1^. It can not only reduce mind-wandering during training, but it can also influence cognitive performance after training, and improve well-being and happiness in daily life^2–4^. Therefore, many mindfulness interventions have been developed as clinical interventions and training programs^1,5^.

Mindfulness meditation and related interventions consist of focused attention meditation (FAM), which improves concentration abilities, and open monitoring meditation (OMM), which improves the ability to monitor the contents of experience without any reactions or judgments^6^. Both FAM and OMM aim to keep the practitioner’s attention away from distractors, such as particular bodily sensations, feelings, and thoughts, which evoke mind-wandering. Consistent with this, FAM and OMM can reduce mind-wandering and activity in the default mode network (DMN), a network associated with mind-wandering^2,7,8^. To achieve this, during FAM, meditators practice sustaining their intentional focused attention on an explicit object. After advancing in FAM, during OMM practitioners reduce intentional focused attention gradually and keep their attention away from distractors without an explicit object^6^.

It can therefore be asked whether FAM and OMM differ only in the strength of intentional focused attention or whether they differ in their function to keep practitioner’s attention away from distractors. Furthermore, it is not clear whether the ability to keep the practitioner’s attention away from distractors is sustained after practicing FAM and OMM.

Although the neural mechanisms underlying FAM overlap with those underlying attention and have been extensively studied^9,10^, little is known about the neural mechanisms underlying OMM because the concept of OMM originates from Buddhism and has just begun to be examined within cognitive neuroscience. The non-reactive and non-judgmental monitoring developed by OMM is an important psychological factor in the improvement of well-being and happiness in daily life^11^. Therefore, it is important to clarify the neural mechanisms underlying OMM and its after-effects.

In FAM, meditators focus their attention on a target object, such as the physical sensations caused by breathing. Having a target object enables meditators to keep their attention away from distractors and to disengage their attention from these distractors more easily^6^. In functional magnetic resonance imaging (fMRI) studies, FAM has been shown to reduce activity in the anterior medial prefrontal cortex (mPFC) and posterior cingulate cortex (PCC)/precuneus^2^, which are core hubs of the DMN^7^. In contrast, increased activation has been observed in brain regions involved in the ventral and dorsal attention network^12^, such as the frontal eye field (FEF), visual cortex, dorsolateral prefrontal cortex (dlPFC), and anterior insula^9^. More specifically, activity in the anterior insula and dorsal anterior cingulate cortex (ACC) increases at moments when meditators realize their mind is wandering during FAM. Subsequently, the recognition of their mind-wandering and movement of their attention to the target object leads to increased activity in the dlPFC, and caudate, and decreased activity in the mPFC^10^. That is, FAM enhances intentional focused attention, which is associated with activity in the attention network.

In OMM, meditators keep a non-reactive and non-judgmental awareness of anything that occurs in their experience of the present moment^6^. While maintaining this awareness, the contents of experience such as bodily sensations, feelings, and thoughts are not distractors but simply contents for observation. However, if meditators react to or judge contents of their experience, as people tend to do automatically and habitually, they become distractors from awareness of the present moment. Compared with FAM, it is more difficult to disengage attention from these distractors during OMM because meditators do not have a target object. Instead, they must simply stop reacting to and judging these distractors. For this purpose, meditators practice OMM to develop a more acute, but less emotionally reactive, awareness of their experiences, including the autobiographical sense of identity that projects back into the past and forward into the future^6^. Although some studies have shown that OMM also reduces activity in the core hubs of the DMN to the same extent as does FAM^2^, precise mechanisms underlying these processes are still unclear. From a theoretical perspective, it is conceivable that OMM reduces memory function involvement in ongoing processes. In fact, Taylor *et al*.^13^ indicated that OMM reduces activity in the hippocampus and retrosplenial cortex (RSC), which are components of the DMN and are associated with self-reference in the past and future^7^.

Furthermore, to understand how changes to cognitive functions during FAM and OMM may influence daily life, it is also important to investigate the after-effects of FAM and OMM. Such after-effects form a bridge between states experienced during meditation and traits in daily life. Some studies have indicated that a short mindfulness intervention also reduces mind-wandering^4^. In this study, we predicted that some alterations in brain activity observed during FAM and OMM would be sustained after meditation. Such brain activity might underlie the effects that FAM and OMM practice have on daily life. Thus, we also investigated the after-effects of brain activity after OMM compared with FAM.

To investigate neural mechanisms underlying OMM and its after-effects compared with FAM, we focused on the attention network and DMN using a functional connectivity analysis, which is a powerful method used to reveal relationships between brain regions. Mindfulness meditation involves a broad range of brain regions, such as the dlPFC, ACC, anterior insula, mPFC, PCC, hippocampus, visual cortex, and striatum^9,14,15^. In particular, the striatum connects to other cortical regions differentially and forms multiple parallel cortical-striatal loops that can be functionally distinguished, such as attention and memory function loops^16–20^. Moreover, the striatum plays an important role in reducing habitual behavior and enhancing self-control, including attention control, emotion regulation, and self-awareness during mindfulness meditation^15,21,22^.

Therefore, to investigate relationships between brain regions underlying self-control and those underlying both attention and memory function, we examined striatal functional connectivity in 17 experienced meditators (mean total practice hours = 920.6) during pre-resting, meditation, and post-resting states comparing OMM with FAM for 6 minutes in each state, using fMRI. In particular, dividing the experiment across two days for FAM and OMM within participants, we were able to allow them 60 minutes for each practice between scans of pre-resting and meditation states. In FAM, participants paid attention to the physical sensation of the breath within a triangle area from the upper lip to nostrils. In contrast, in OMM, participants observed whatever bodily sensation came into their awareness non-reactively and non-judgmentally. In addition, prior to each scanning day, participants had meditated for 30 minutes in their home for seven days following the same instructions as in the experiment.

The main aim of this study was to elucidate the striatal functional connectivity with both attention network regions and DMN regions during OMM by comparing it with functional connectivity during FAM. We hypothesized that OMM would differ from FAM, not only in the intentional focused attention, but also in the involvement of memory function. That is, during FAM, in response to increasing intentional focused attention, the relationship between the striatum and attention network regions would increase. In contrast, if OMM does not require intentional focused attention, during OMM the relationship between the striatum and attention network regions would decrease. Furthermore, in response to decreased involvement of memory function, the relationship between the striatum and DMN regions related to memory function would also decrease. In addition, there would be correlations between such changes during meditation and meditation practice hours. The second aim of this study was to elucidate the after-effects of FAM and OMM. Therefore, we conducted an investigation into after-effects concentrating on functional connectivity that showed changes during FAM or OMM.

## Results

### Practicing each meditation technique

All but one participant practiced FAM and OMM at home for seven days. One participant practiced OMM at home for just five days, but practiced FAM for seven days. Since this participant had enough understanding of how to do OMM, we did not exclude their data from further analyses.

### Self-report

There were no significant differences between how well FAM was performed during 60 minutes in a soundproof chamber (mean = 3.2, SD = 0.5) and during 6 minutes in an MRI scanner (mean = 3.3, SD = 0.6) [t (16) = 0.17, p = 0.86]. The results were the same for OMM (soundproof chamber (mean = 3.4, SD = 0.6) and MRI scanner (mean = 3.3, SD = 0.5) [t (16) = 1.28, p = 0.22]). Although fMRI scanning was very noisy and participants were laying down, they could subjectively perform FAM and OMM as well as they could while sitting in the calm room. During the post-resting state, 14 participants reported that they were able to stop FAM while the others reported that they could not determine whether they stopped FAM or not. Twelve participants reported that they were able to stop OMM during the post-resting state, while the others reported they could not determine whether they stopped OMM or not. This suggests that all participants did not meditate, at least intentionally, during the post-resting state.

### Functional connectivity during FAM and OMM

We investigated functional connectivity differences between the pre-resting and meditation states for each meditation condition, using six bilateral regions of interest (ROIs) consisting of 3.5 mm radius spheres centered on MNI coordinates from a previous study^23^. The ROIs were as follows: ventral caudate (inferior)/nucleus accumbens (VSi), ventral caudate (superior) (VSs), dorsal caudate (DC), dorsal caudal putamen (DCP), dorsal rostral putamen (DRP), and ventral rostral putamen (VRP) (Table 1). Tables 2 and 3 (for FAM) and Tables 4 and 5 (for OMM) list all regions in which significant changes were observed.

**Table 1:** Coordinates for striatal regions of interest.

		X	Y	Z
Ventral Caudate (inferior)	VSi	(±) 9	9	−8
Ventral Caudate (superior)	VSs	(±) 10	15	0
Dorsal Caudate	DC	(±) 13	15	9
Dorsal Caudal Putamen	DCP	(±) 28	1	3
Dorsal Rostral Putamen	DRP	(±) 25	8	6
Ventral Rostral Putamen	VRP	(±) 20	12	−3

Coordinates for the right and left hemisphere seeds are defined in the MNI stereotaxic space.

**Table 2:** Increased functional connectivity between ROIs and other brain regions during FAM compared with the pre-resting state.

ROI	MNI			Correlation		T	Voxels		Brain region (BA)	AE
	x	y	z	Rest	FAM		total	detail		
l_DRP	26	0	6	0.31**	0.51**	5.69	169	81 20	r_putamen r_pallidum	— —

Results from a comparison between the pre-resting state and meditation state (*p* < 0.001 uncorrected, k ≥ 10, cluster FWE < 0.001). Correlation indicates functional connectivity between the ROI and other brain regions during the pre-resting state and meditation state (**p < 0.01). T indicates peak T-values. For the Voxels (voxels per cluster) column, only clusters of 10 or more voxels are shown. After-effect (AE) indicates differences in correlations between the pre-resting and post-resting states. BA: Brodmann area; l: left; r: right; DRP: dorsal rostral putamen.

**Table 3:** Decreased functional connectivity between ROIs and other brain regions during FAM compared with the pre-resting state.

ROI	MNI			Correlation		T	Voxels		Brain region (BA)	AE
	x	y	z	Rest	FAM		total	detail		
l_DCP	22	−56	4	0.06	−0.12**	5.13	320	118 68 45 26 15 12 12	r_RSC (30) r_secondary VC (18) r_primary VC (17) r_ventral PCC (23) r_associative VC (19) l_RSC (30) r_dorsal PCC (31)	† † † † † — —
r_DRP	2	−26	44	0.03	−0.10**	7.00	200	126 40 21	l_dorsal PCC (31) l_ventral precuneus (7) r_dorsal PCC (31)	— — —

Results from a comparison between the pre-resting state and meditation state (*p* < 0.001 uncorrected, k ≥ 10, cluster FWE < 0.001). Correlation indicates functional connectivity between the ROI and other brain regions during the pre-resting state and meditation state (**p < 0.01). T indicates peak T-values. For the Voxels (voxels per cluster) column, only clusters of 10 or more voxels are shown. After-effect (AE) indicates differences in correlations between the pre-resting and post-resting states (^†^*p* < 0.001 uncorrected, k ≥ 10, small volume collection). BA: Brodmann area; l: left; r: right; DCP: dorsal caudal putamen; DRP: dorsal rostral putamen; RSC: retrosplenial cortex; VC: visual cortex; PCC: posterior cingulate cortex.

**Table 4:** Increased functional connectivity between ROIs and other brain regions during OMM compared with the pre-resting state.

ROI	MNI			Correlation		T	Voxels		Brain region (BA)	AE
	x	y	z	Rest	OMM		total	detail		
r_VSs	−60	−58	−6	−0.13**	0.07	5.96	293	146 105 12	l_MTG (21) l_fusiform gyrus (37) l_STG (22)	† — †

Results from a comparison between the pre-resting state and meditation state (*p* < 0.001 uncorrected, k ≥ 10, cluster FWE < 0.001). Correlation indicates functional connectivity between the ROI and other brain regions during the pre-resting state and meditation state (**p < 0.01). T indicates peak T-values. For the Voxels (voxels per cluster) column, only clusters of 10 or more voxels are shown. After-effect (AE) indicates differences in correlations between the pre-resting and post-resting states (^†^*p* < 0.001 uncorrected, k ≥ 10, small volume collection). BA: Brodmann area; l: left; r: right; VSs: ventral caudate (superior); MTG: middle temporal gyrus; STG: superior temporal gyrus.

**Table 5:** Decreased functional connectivity between ROIs and other brain regions during OMM compared with the pre-resting state.

ROI	MNI			Correlation		T	Voxels		Brain region (BA)	AE
	x	y	z	Rest	OMM		total	detail		
r_VSi	−32	−80	−8	0.10**	−0.07*	6.53	247	102 96	l_associative VC (19) l_secondary VC (18)	— —
r_VSs	30	36	38	0.12**	−0.08*	8.33	534	348 63 20 13	r_FEF (8) r_dlPFC (9) r_premotor cortex (6) r_dorsal ACC (32)	† — † —
	−30	30	32	0.09**	−0.06*	8.19	346	123 66 44 22	l_FEF (8) l_dlPFC (9) l_premotor cortex (6) l_dorsal ACC (32)	— — — —
	10	32	0	0.24**	0.05	5.72	187	65 41 15	r_dorsal ACC (32) r_ventral ACC (24) r_anterior mPFC (10)	— — —
l_DCP	54	−46	−2	0.10**	−0.10**	10.73	673	120 69 56	r_fusiform gyrus (37) r_MTG (21) r_ITG (20)	† — —
	−24	−38	−6	0.08*	−0.09**	6.24	207	75 36 22	l_RSC (30) l_PHG (36) l_associative VC (19)	— — —
r_DCP	54	−48	−4	0.11**	−0.08**	6.64	217	41	r_fusiform gyrus (37)	†
l_DRP	40	20	−36	0.07**	−0.12**	6.48	232	193	r_TP (38)	—
	12	62	20	0.09**	−0.07**	7.36	211	201	r_dlPFC (9)	—
r_DRP	24	−34	−4	0.05**	−0.12**	5.84	304	165 39 27	r_RSC (30) r_piriform cortex (27) r_RSC (29)	† † —
	−16	−48	0	0.01	−0.14**	5.81	170	79 53 15	l_RSC (30) l_associative VC (19) l_secondary VC (18)	— — —
	38	−72	26	0.08*	−0.11**	5.01	160	11 10	r_associative VC (19) r_dorsal PCC (31)	— —
l_VRP	46	−56	16	0.09**	−0.07**	7.23	443	105 76 41 19 14 11	r_STG (22) r_angular gyrus (39) r_associative VC (19) r_insular cortex (13) r_SMG (40) r_primary auditory cortex (41)	— — — — — —
	18	−46	4	0.10**	−0.07**	5.50	342	191 40 20 15	r_RSC (30) r_secondary VC (18) r_RSC (29) r_associative VC (19)	— — — —

Results from a comparison between the pre-resting state and meditation state (*p* < 0.001 uncorrected, k ≥ 10, cluster FWE < 0.001). Correlation indicates functional connectivity between the ROI and other brain regions during the pre-resting state and meditation state (*p < 0.05; **p < 0.01). T indicates peak T-values. For the Voxels (voxels per cluster) column, only clusters of 10 or more voxels are shown. After-effect (AE) indicates differences in correlations between the pre-resting and post-resting states (^†^*p* < 0.001 uncorrected, k ≥ 10, small volume collection). BA: Brodmann area; l: left; r: right; VSi: ventral caudate (inferior); VSs: ventral caudate (superior); DCP: dorsal caudal putamen; DRP: dorsal rostral putamen; VRP: ventral rostral putamen; VC: visual cortex; FEF: frontal eye field; dlPFC: dorsolateral prefrontal cortex; ACC: anterior cingulate cortex; mPFC: medial prefrontal cortex; MTG: middle temporal gyrus; ITG: inferior temporal gyrus; RSC: retrosplenial cortex; PHG: parahippocampal gyrus; TP: temporal pole; PCC: posterior cingulate cortex; STG: superior temporal gyrus; SMG: supramarginal gyrus.

During FAM compared with the pre-resting state, no increased connectivity was observed between the striatum and brain regions in the attention network and DMN. In contrast, there were decreased connectivity of the dorsal putamen with both the visual cortex and DMN regions, including the PCC/precuneus and RSC.

During OMM compared with the pre-resting state, there were increased connectivity of the ventral striatum with the several DMN regions, including the middle temporal gyrus (MTG) and superior temporal gyrus (STG). No increased connectivity was observed between the striatum and core hub regions of the DMN. In contrast, there were decreased connectivity with attention network regions, including between the ventral striatum and the dlPFC, FEF, and visual cortex, and between the putamen and the dlPFC, anterior insula, supramarginal gyrus (SMG), and visual cortex. Furthermore, there was decreased connectivity between the putamen and many DMN regions, which included not only the PCC but also memory-related regions, such as the RSC, parahippocampal gyrus, and piriform cortex.

### Direct comparisons of functional connectivity between FAM and OMM

**Figure 1:**
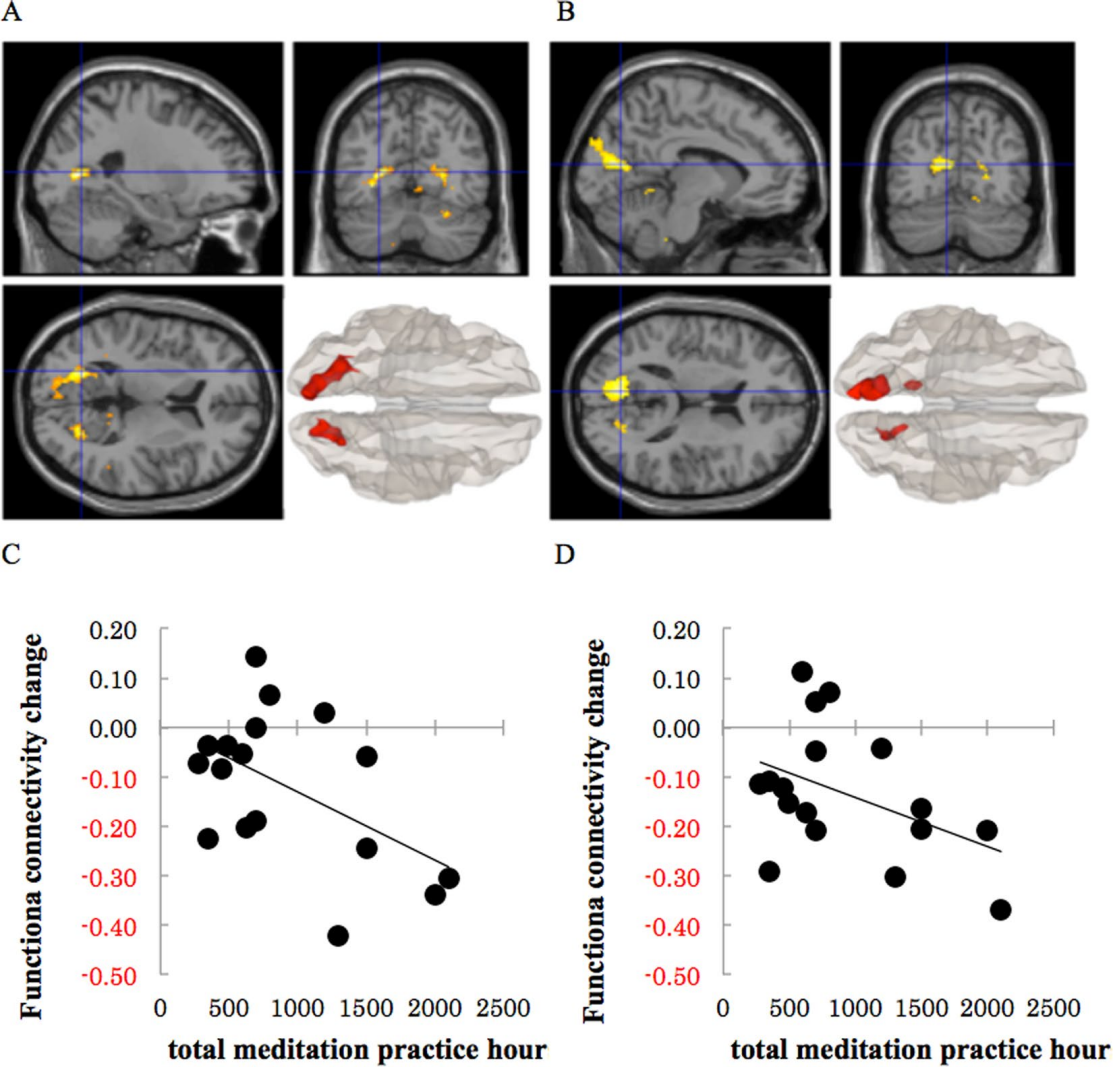
Results from an investigation of the interaction between timing and meditation (**A**) indicates the results of a functional connectivity analysis of the left ventral striatum inferior. (**B**) indicates a functional connectivity analysis of the left ventral rostral putamen. (**C**) indicates the correlation between meditation practice hours and altered functional connectivity from the resting state to the open monitoring meditation (OMM) state between the left ventral rostral putamen and left retrosplenial cortex (−24, −66, 8). (**D**) indicates the correlation between meditation practice hours and altered functional connectivity from the resting state to the OMM state between the left ventral rostral putamen and right retrosplenial cortex (18, −68, 14).

We investigated the interaction between timing (pre-resting and meditation states) and meditation (FAM and OMM). Table 6 lists all regions in which a significant interaction was observed. There was increased connectivity of the left VSi and left VRP with attention network regions, including the visual cortex, and DMN regions, including the PCC and RSC from the pre-resting state to the FAM state. Meanwhile, there was decreased connectivity of the left VSi and left VRP with the visual cortex, PCC, and RSC from the pre-resting state to the OMM state (Fig. 1A,B). There was no decreased connectivity from the pre-resting state to the FAM state or increased connectivity from the pre-resting state to the OMM state.

**Table 6:** Interaction of changing from the pre-resting state to the meditation state between FAM and OMM.

ROI	MNI			Simple Main effect		T	Voxels		Brain regions (BA)	AE
	x	y	z	FAM	OMM		total	detail		
rest < FAM, rest > OMM										
l_VSi	−8	−72	14	§	§§	5.67	483	192 89 80 48 26	l_secondaryVC (18) l_primary VC (17) l_RSC (30) l_ventral PCC (23) l_associative VC (19)	— — — — —
l_VRP	−24	−66	8	§	§§	7.56	559	174 133 63 33	l_primary VC (17) l_secondary VC (18) l_RSC (30) l_associative VC (19)	— — — —
	18	−68	14	−	§§	7.10	287	79 73 62 15 14	r_primary VC (17) r_RSC (30) r_secondary VC (18) r_ventral PCC (23) r_associative VC (19)	— — — — —

Results represent a comparison from the pre-resting state to the meditation state between FAM and OMM (*p* < 0.001 uncorrected, k ≥ 10, cluster FWE < 0.001). The simple main effect column indicates details for the simple main effects of the interaction (^§^*p* < 0.05; ^§§^*p* < 0.001). T indicates peak T-values. For the Voxels (voxels per cluster) column, only clusters of 10 or more voxels are shown. After-effect (AE) indicates the differences in correlations between the pre-resting and post-resting states. BA: Brodmann area; l: left; r: right; VSi: ventral caudate (inferior); VRP: ventral rostral putamen; VC: visual cortex; RSC: retrosplenial cortex; PCC: posterior cingulate cortex.

Moreover, we observed a significant correlation between total meditation practice hours and functional connectivity changes from the pre-resting state to the OMM state between the left VRP and left RSC (*r* = −0.52, *p* = 0.03) (Fig. 1C). In addition, there was also an approached significance correlation between total meditation practice hours and functional connectivity changes from the pre-resting state to the OMM state between the left VRP and left RSC (*r* = −0.43, *p* = 0.09) (Fig. 1D).

### After-effects of FAM and OMM

We investigated functional connectivity differences between the pre-resting and post-resting states for each meditation condition within regions that demonstrated significant connectivity changes from the pre-resting state to the meditation state in the first analysis. Table 3 and SI Table 1 (for FAM), and Tables 4, 5, and SI Tables 2 and 3 (for OMM) list all regions in which significant after-effects were observed. During FAM, decreased connectivity between the DCP and both the visual cortex and DMN regions, including the PCC and RSC, was sustained. On the other hand, during OMM, increased connectivity between the ventral striatum and both the MTG and STG was sustained. In contrast, decreased connectivity between the DRP and DMN regions, including the RSC and piliform cortex, was sustained.

### Direct comparison of after-effects between FAM and OMM

We investigated the interaction between timing (pre-resting and post-resting states) and meditation (FAM and OMM) within regions that demonstrated a significant interaction between timing (pre-resting and meditation states) and meditation (FAM and OMM) in the second analysis. We could not find any significant maintenance of the interaction of functional connectivity.

## Discussion

In this study, we first confirmed the hypothesis that OMM would differ from FAM, not only in the intentional focused attention, but also in the involvement of memory function by investigating striatal functional connectivity with both attention network regions and DMN regions. We observed that both meditation techniques reduced connectivity between the striatum and core hub regions of the DMN that are related to mind-wandering. Furthermore, there were significant interactions between timing and meditation. OMM decreased connectivity between the striatum and both the visual cortex in the attention network and RSC in the DMN related to memory function, while FAM increased striatal functional connectivity with the visual cortex and RSC. These results support our hypothesis. Subsequently, we investigated after-effects of connectivity changes observed during FAM or OMM. Although we did not find sustained connectivity related to significant interactions mentioned above, we did observe that some connectivity changes were sustained from the pre-resting state to the meditation state.

Meditators in our study reported that they could do both FAM and OMM in the scanner as well as in the calm room. Furthermore, we observed a clear difference in functional connectivity between FAM and OMM. Taken together, this experimental design appears to be a valid method to successfully extract brain activity underlying FAM and OMM.

Our findings regarding striatal functional connectivity during the pre-resting state are consistent with a previous study^23^, although there were a few differences. In the previous study, among DMN regions negative correlations were reported between the right DRP and right dorsal PCC, and between the left VRP and right STG during the resting state, whereas in our study these correlations were weakly positive. Since connectivity between the striatum and DMN regions gradually changes from negative to positive depending on age^24^, these differences might be because the mean age of our participants was older than that of the participants of the previous study.

To obtain an overview of functional connectivity for each type of meditation, we investigated differences in correlations between the pre-resting and meditation states for each meditation condition within participants. During both FAM and OMM, compared with the pre-resting state, there was decreased connectivity of the dorsal putamen with core hub regions of the DMN. Furthermore, both FAM and OMM decreased connectivity between the putamen and DMN regions from a positive to negative correlation. In particular, decreased connectivity during OMM included memory-related brain regions. Although the physiological mechanisms underlying negative correlations remain controversial, some previous studies have indicated that negative connectivity between the putamen and core hub regions of the DMN might be associated with modulation of activity in DMN regions^19,25^. Given that both FAM and OMM reduce mind-wandering and activity in DMN regions^2^, our findings are consistent with those of previous studies.

During FAM compared with OMM, there was increased functional connectivity of the left VRP with the visual cortex. Although the function of such connectivity is unclear, one interpretation is that this connectivity is associated with intentional focused attention with an explicit target object. The visual cortex is related to selective attention^12^, and the visual cortex and putamen show co-activation during a task that demands an attentional switch from internal thoughts to an external target^26^. Furthermore, experienced meditators compared with novices, showed co-activation of these regions during FAM^9^. In the present study, functional connectivity among such attention regulation regions increased during FAM and decreased during OMM. Given that FAM requires intentional focused attention with the use of an explicit object, and that OMM requires gradual reduction of intentional focused attention and maintaining attention away from a distractor without an explicit object^6^, increased connectivity between the putamen and the visual cortex appears to be associated with intentional focused attention regulation with an explicit object.

During OMM compared with FAM, there was decreased functional connectivity of both the left VRP and left VSi with the RSC. The RSC is part of the PCC and receives major inputs from the orbital prefrontal cortex, dlPFC, ACC, hippocampus, and precuneus, playing a crucial role in autobiographical memory^27^. Previous fMRI studies indicated that the RSC is activated by the retrieval of autobiographical memory^28–30^, and it is associated with the integration of information from the hippocampus with a first-person perspective^29^. Furthermore, a correlation between activation of the RSC and self-report of how much an individual was able to re-experience an event during autobiographical memory retrieval has been reported^28^. Given that OMM improves the ability to detach from experiences such as autobiographical memories^6^, the RSC might play an important role.

Furthermore, the putamen is a core hub region within a strongly interconnected structural brain network along with other regions such as the hippocampus and precuneus^20^. Given that the putamen is activated during retrieval of personal highly stressful life events in conjunction with PCC and hippocampus activation^31,32^, this region is likely to be another core hub of the network involved in self-reference related to autobiographical memory retrieval. In addition, the VSi plays an important role in reward and motivation^33^. In particular, increased functional connectivity of the VSi with the RSC during a resting state is associated with addiction and habitual behavior, such as drug addiction and internet addiction^34,35^. Based on the above-mentioned idea that negative connectivity between the putamen and DMN regions might also be associated with modulation of DMN activity, one interpretation of decreased connectivity of both the VRP and VSi with the RSC is that it is associated with detachment from autobiographical memory without reacting to or judging experiences automatically and habitually.

In addition, given that participants usually practice OMM in daily life, the correlation between meditation practice hours and decreased functional connectivity indicates that these connectivity patterns are characteristic of OMM. This is in accordance with previous research that has indicated that experienced meditators compared with beginners show decreased activation of the RSC during OMM^13^.

After FAM compared with the pre-resting state, we observed sustained decreased functional connectivity between the DCP and both the ventral PCC and RSC, although all participants reported they did not meditate, at least intentionally, during the post-resting state. Furthermore, after OMM compared with the pre-resting state, decreased connectivity between the DRP and both the RSC and piriform cortex was sustained, although all participants reported they did not meditate, at least intentionally, during the post-resting state. This sustained connectivity implies that the effect of meditation related to the modulation of DMN activity persists for some time after meditation, which is consistent with previous studies indicating that brief FAM and OMM alters performance on attention and memory tasks following the meditation session^4,36,37^. Further research is needed to clarify the relationship between these sustained effects and task performance.

## Conclusions

In conclusion, this research demonstrates that OMM has different effects to FAM on the striatal functional connectivity with both attention network regions and DMN regions. Taking the theoretical aspects of OMM into consideration, our findings suggest that functional connectivity changes during OMM may be associated with reduced intentional focused attention and increased detachment from autobiographical memory without reacting to or judging experiences automatically and habitually.

## Methods

### Participants

Eighteen right-handed meditators (7 females, 11 males; mean age = 33.5, SD = 8.0; including male author M.F.) were recruited from a local Vipassana meditation center. All had participated in a 10-day intensive meditation retreat at least once (mean number of attendances = 3.6, SD = 2.7), and kept daily meditation practices (mean total practice hours = 1,147.2, SD = 1,111.0). Participants had practiced both FAM and OMM at the 10-day retreat. In daily life, they practiced OMM regularly but practiced FAM when they were not able to regulate their attention well.

One male participant from this group had experience of 5,000 hours of meditation practice. Therefore, he deviated in the number of hours of experience from the rest of the group (7 females, 10 males; mean age = 32.7, *SD* = 7.5; mean total practice hours = 920.6, *SD* = 573.7). Although the results remained essentially unchanged with the inclusion of this participant, we excluded this participant from the statistical analyses, because we aimed to investigate the relationship between the meditation practice hours and functional connectivity.

Before beginning, written instructions were given and written informed consent was obtained from all participants. All procedures were approved by the internal ethics committees of the Graduate School of Human and Environmental Studies, and Graduate School of Education at Kyoto University. All methods were performed in accordance with the relevant guidelines and regulations.

### Task and procedures

Participants completed FAM and OMM sessions on two different days within two weeks. The order of FAM and OMM sessions was counterbalanced across participants. One week before each session, participants were given the FAM or OMM instructions used in this study and asked to practice. Practice required them to meditate with eyes open for 30 minutes every day for one week. We used a modified version of instructions from Brewer *et al*.^2^. For FAM, participants were instructed: “Please open your eyes, and pay attention to the physical sensation of the breath within the triangle area from the upper lip to nostrils. Follow the natural and spontaneous movements of the breath, not trying to change it in any way. Just pay attention to it. If you find that your attention has wandered to something else, gently but firmly bring it back to the physical sensation of the breath within the triangle area.” For OMM, participants were instructed: “Please open your eyes, and pay attention to whatever bodily sensation comes into your awareness. Just follow it until other bodily sensations come into your awareness, not trying to hold on to them or change them in any way. When other bodily sensations come into your awareness, just pay attention to them until the next comes.”

Before each session on the experimental day, participants were given the meditation instructions again. In each session, there were three fMRI scans of 6 minutes: during rest before meditation (pre-resting state), during meditation (meditation state), and during rest after meditation (post-resting state). During rest, participants were instructed: “Please open your eyes and relax yourself. Don’t think of anything in particular.” Between the pre-resting state and meditation state, participants meditated for 60 minutes in a soundproof chamber next to the scanner room to achieve a deep meditative state. Between the meditation state and post-resting state, they exited the scanner and stretched for 3 minutes to stop meditating before the post-resting state. After the fMRI scan, participants rated how well they were able to meditate for 60 minutes in a soundproof chamber and for 6 minutes in the scanner on a scale from 1 to 5 (1: not at all; 5: very well). Furthermore, they also rated whether they were able to stop meditating during the post-resting state on a scale of 1 to 3 (1: I was able to stop meditating; 2: neither; 3: I was not able to stop meditating).

### MRI acquisition

MRI data were acquired using a 3T Verio (Siemens) located at Kokoro Research Center, Kyoto University. Structural data were acquired with a high-resolution magnetization prepared rapid acquisition gradient echo T1-weighted sequence (208 axial slices; no gap between slice acquisition; repetition time (TR) = 2250 ms; echo time (TE) = 3.51 ms; field of view = 256 mm; matrix = 256 × 256; voxel size: 1.0 × 1.0 × 1.0 mm; flip angle = 9°). Functional data were acquired with a gradient-echo echo-planar imaging sequence (34 axial slices; no gap between slice acquisition; TR = 2000 ms; TE = 25 ms; field of view = 224 mm; matrix = 64 × 64; voxel size: 3.5 × 3.5 × 3.5 mm; flip angle = 75°).

### MRI pre-processing

MRI data were pre-processed with SPM 12 (Wellcome Department of Cognitive Neurology, London, UK). The first 10 volumes of each run of functional data were excluded from the analyses to achieve signal stability. The functional data were slice-time corrected to the middle slice of each volume and realigned to the first volume with a six-parameter rigid body transformation. The structural data were normalized onto the common stereotactic reference space of the Montreal Neurological Institute (MNI), and were segmented into gray matter, white matter, and cerebrospinal fluid (CSF), and were extracted from the skull. Functional data were then coregistered to structural data and were smoothed with a 6 mm full-width half-maximum isotropic Gaussian kernel.

### Functional connectivity analyses

Functional connectivity analyses were performed with the CONN-fMRI Functional Connectivity toolbox v15.g^38^. To investigate striatal functional connectivity, we selected six bilateral ROIs consisting of 3.5 mm radius spheres centered on MNI coordinates from a previous study^23^. The ROIs were as follows: ventral caudate (inferior)/nucleus accumbens (VSi), ventral caudate (superior) (VSs), dorsal caudate (DC), dorsal caudal putamen (DCP), dorsal rostral putamen (DRP), and ventral rostral putamen (VRP) (Table 1). Using denoising processing, the confounding effects of head motion, and BOLD signals from white matter and CSF were removed. The residual BOLD time series were also band-pass filtered from 0.01 Hz to 0.1 Hz.

In the first-level analyses, an individual participant’s seed-to-voxel connectivity maps for six states (timing: pre-resting, meditation, and post-resting, and meditation condition: FAM and OMM) were created for the six bilateral ROIs. The mean BOLD time series was computed across all voxels within each ROI. Bivariate correlation analyses were used to calculate the Fisher-transformed correlation coefficients of the BOLD time series between each pair of source ROI and target voxel. Each scan was HRF-weighted^38^.

Individual seed-to-voxel maps were used in the second-level analysis, in which age was included as a covariate. We conducted four types of analyses. First, we conducted an analysis to obtain an overview of striatal functional connectivity for each type of meditation. Therefore, we investigated correlation differences between the pre-resting and meditation states for each meditation condition within participants. The threshold for significant changes was set at *p* < 0.001 for voxel level, uncorrected, *k* ≥ 10, and at *p* < 0.001 for cluster level, FWE corrected for multiple comparisons^39^. To investigate whether each correlation was significantly different from zero, we conducted one-sample *t*-tests with significance indicated by *p* < 0.05.

Second, we conducted an analysis to elucidate differences in striatal functional connectivity during FAM and OMM. Therefore, we investigated the interaction between timing (pre-resting and meditation states) and meditation (FAM and OMM) with the same threshold as for the first analysis. Furthermore, we also calculated the correlation between total meditation practice hours and connectivity changes from the pre-resting state to the meditation state.

Third, we conducted an analysis to obtain an overview of the after-effects of each type of meditation. As such, we investigated correlation differences between the pre-resting and post-resting states for each meditation condition within regions in which functional connectivity changes were significant in the first analysis. The threshold for significant changes was set at *p* < 0.001 for voxel level, uncorrected, *k* ≥ 10, and small volume corrected.

Finally, we conducted an analysis to clarify the differences in after-effects between FAM and OMM. Therefore, we investigated the interaction between timing (pre-resting and post-resting states) and meditation (FAM and OMM) within regions in which functional connectivity changes were significant in the second analysis with the same threshold as the third analysis.

### Data availability

The authors declare that the data of the study is available.

## Electronic supplementary material

Supplementary info
