# Supplementary material for: Open monitoring meditation reduces the involvement of brain regions related to memory function
Source: Sci Rep. 2018 Jul 2;8:9968. doi: 10.1038/s41598-018-28274-4 (PMC6028418; doi:10.1038/s41598-018-28274-4)
Supplement: Supplementary file 1 — Supplementary info [file 41598_2018_28274_MOESM1_ESM.docx]

*Title*

Open monitoring meditation reduces the involvement of brain regions related to memory function

*Author names and affiliations*

Masahiro Fujino^a, e,^ *, Yoshiyuki Ueda^b,^ *, Hiroaki Mizuhara^c^, Jun Saiki^d^, and Michio Nomura^a^

^a^ Graduate School of Education, Kyoto University, Yoshida-Honmachi, Sakyo-ku, Kyoto-shi, Kyoto, 606-8501, Japan.

^b^ Kokoro Research Center, Kyoto University, 46 Shimoadachi-cho, Yoshida, Sakyo-ku, Kyoto-shi, Kyoto, 606-8501, Japan.

^c^ Graduate School of Informatics, Kyoto University, Yoshida-Honmachi, Sakyo-ku, Kyoto-shi, Kyoto, 606-8501, Japan.

^d^ Graduate School of Human and Environmental Studies, Kyoto University, Yoshida-Honmachi, Sakyo-ku, Kyoto-shi, Kyoto, 606-8501, Japan.

^e^ Japan Society for the Promotion of Science, 5-3-1, Kojimachi, Chiyoda-ku, Tokyo, Japan, 102-0083

* Corresponding authors

*Contact information*

Masahiro Fujino

E-mail: fujino.masahiro.68a@kyoto-u.jp

Yoshiyuki Ueda

E-mail: ueda.yoshiyuki.3e@kyoto-u.jp

Supplemental information

SI Table 1. Sustained effect of decreased functional connectivity after FAM

| ROI | MNI | | | Correlation | | T | Voxels | Brain region (BA) |
| --- | --- | --- | --- | --- | --- | --- | --- | --- |
|  | x | y | z | pre-Rest | post-Rest |  | total |  |
| l_DCP | 6 | -82 | 14 | 0.05__ | -0.13** | 6.80 | 89 | r_RSC (30) r_secondary VC (18) r_primary VC (17) r_ventral PCC (23) |
|  | 18 | -58 | 8 | 0.04__ | -0.16** | 4.87 | 83 | r_RSC (30) r_secondary VC (18) r_associative VC (19) |

Results from a comparison between the pre-resting and post-resting states within regions in which functional connectivity changes were significant in the first analysis (*p* < 0.001 uncorrected, k ≥ 10, small volume collection). Correlation indicates functional connectivity between the ROI and other brain regions during the pre-resting and post-resting states (** *p* < 0.01). T indicates peak T-values. ROI: region of interest; BA: Brodmann area; l: left; r: right; DCP: dorsal caudal putamen; RSC: retrosplenial cortex; VC: visual cortex; PCC: posterior cingulate cortex.

SI Table 2. Sustained effect of increased functional connectivity after OMM

| ROI | MNI | | | Correlation | | T | Voxels | Brain region (BA) |
| --- | --- | --- | --- | --- | --- | --- | --- | --- |
|  | x | y | z | pre-Rest | post-Rest |  | total |  |
| r_VSs | -64 | -50 | 12 | -0.12** | 0.04__ | 5.12 | 62 | l_MTG (21) l_STG (22) |

Results from a comparison between the pre-resting and post-resting states within regions in which functional connectivity changes were significant in the first analysis (*p* < 0.001 uncorrected, k ≥ 10, small volume collection). Correlation indicates functional connectivity between the ROI and other brain regions during the pre-resting and post-resting states (** *p* < 0.01). T indicates peak T-values. ROI: region of interest; BA: Brodmann area; l: left; r: right; VSs: ventral caudate (superior); MTG: middle temporal gyrus; STG: superior temporal gyrus.

SI Table 3. Sustained effect of decreased functional connectivity after OMM

| ROI | MNI | | | Correlation | | T | Voxels | Brain region (BA) |
| --- | --- | --- | --- | --- | --- | --- | --- | --- |
|  | x | y | z | pre-Rest | post-Rest |  | total |  |
| r_VSs | 28 | 42 | 48 | 0.13** | -0.04__ | 7.11 | 172 | r_FEF (8) r_premotor cortex (6) |
| l_DCP | 50 | -44 | -16 | 0.11** | -0.05*_ | 5.25 | 123 | r_fusiform gyrus (37) |
| r_DCP | 52 | -48 | -12 | 0.11** | -0.04__ | 6.70 | 93 | r_fusiform gyrus (37) |
| r_DRP | 20 | -32 | -2 | 0.13** | -0.04*_ | 5.85 | 40 | r_RSC (30) r_piriform cortex (27) |

Results from a comparison between the pre-resting and post-resting states within regions in which functional connectivity changes were significant in the first analysis (*p* < 0.001 uncorrected, k ≥ 10, small volume collection). Correlation indicates functional connectivity between the ROI and other brain regions during the pre-resting and post-resting states (* *p* < 0.05; ** *p* < 0.01). T indicates peak T-values. ROI: region of interest; BA: Brodmann area; l: left; r: right; VSs: ventral caudate (superior); DCP: dorsal caudal putamen; DRP: dorsal rostral putamen; FEF: frontal eye field; RSC: retrosplenial cortex.
